# Unorthodox localization of P2X7 receptor in subcellular compartments of skeletal system cells

**DOI:** 10.3389/fcell.2023.1180774

**Published:** 2023-05-04

**Authors:** Letizia Penolazzi, Maria Pina Notarangelo, Elisabetta Lambertini, Valentina Vultaggio-Poma, Mario Tarantini, Francesco Di Virgilio, Roberta Piva

**Affiliations:** ^1^ Department of Neuroscience and Rehabilitation, University of Ferrara, Ferrara, Italy; ^2^ Department of Medical Sciences, University of Ferrara, Ferrara, Italy

**Keywords:** P2X7 receptor, subcellular localization, purinergic signaling, immunogold and electron microscopy, osteoblasts, chondrocytes, intervertebral disc cells

## Abstract

Identifying the subcellular localization of a protein within a cell is often an essential step in understanding its function. The main objective of this report was to determine the presence of the P2X7 receptor (P2X7R) in healthy human cells of skeletal system, specifically osteoblasts (OBs), chondrocytes (Chs) and intervertebral disc (IVD) cells. This receptor is a member of the ATP-gated ion channel family, known to be a main sensor of extracellular ATP, the prototype of the danger signal released at sites of tissue damage, and a ubiquitous player in inflammation and cancer, including bone and cartilaginous tissues. Despite overwhelming data supporting a role in immune cell responses and tumor growth and progression, a complete picture of the pathophysiological functions of P2X7R, especially when expressed by non-immune cells, is lacking. Here we show that human wild-type P2X7R (P2X7A) was expressed in different samples of human osteoblasts, chondrocytes and intervertebral disc cells. By fluorescence microscopy (LM) and immunogold transmission electron microscopy we localized P2X7R not only in the canonical sites (plasma membrane and cytoplasm), but also in the nucleus of all the 3 cell types, especially IVD cells and OBs. P2X7R mitochondrial immunoreactivity was predominantly detected in OBs and IVD cells, but not in Chs. Evidence of subcellular localization of P2X7R may help to i. understand the participation of P2X7R in as yet unidentified signaling pathways in the joint and bone microenvironment, ii. identify pathologies associated with P2X7R mislocalization and iii. design specific targeted therapies.

## Introduction

It is well known that function and stability of intracellular proteins are critically determined by their subcellular localization. Each cell compartment offers different biochemical environments, is exposed to different mechanical triggers and allows interaction with different partners, which altogether contribute to a fine-tuned mechanism of control of intracellular protein localization (Bauer et al., 2015).

A broad variety of biological functions, including protein secretion, cell growth and repair, intracellular signal propagation, establishment of organelle contact, energy metabolism and spatial organization of metabolic gradients, involve protein movement across different cell compartments (Itzhak et al., 2016; Mogre et al., 2020). Changing the location of a protein can modify its activity, which will modulate its functions and affect its fate. At the same time, subcellular mis-localization of certain proteins is correlated with the onset of specific human pathologies, and may be a hallmark of disease (Andrejew et al., 2020; Kumar and Lapierre, 2021; Yousefi et al., 2021). Surprisingly, analysis of changes of subcellular protein localization is currently much under investigated compared to the mere study of their expression levels. For some proteins, more than others, investigation of the subcellular localization under different pathophysiological conditions may represent a real challenge. This is the case of the P2X7 receptor (P2X7R), an ATP-gated plasma membrane ion channel (Adinolfi et al., 2005). Upon activation by extracellular ATP (eATP), the P2X7R generates a non-selective channel permeable to mono- and di-valent cations (Na^+^, Ca^2+^ influx, and K^+^ efflux) (Di Virgilio et al., 2018a). Depending on its activation state, P2X7R can either drive cell survival and proliferation, or induce cell death (Lara et al., 2020). The prolonged activation of P2X7R by high levels of eATP over an extended time period can lead to the formation of a macropore allowing transmembrane fluxes of molecules up to 900 Da, promoting plasma membrane depolarization, and ultimately cell death (Di Virgilio et al., 2018b). No doubt, cytotoxicity is the more widely accepted function associated to the P2X7R. Over the last years however, it has also become clear that this receptor is one of the most potent activators of the NLRP3 inflammasome, and therefore a strong promoter of inflammation in different areas including joint, lung and eye (Di Virgilio et al., 2017; Platania et al., 2022). Finally, a role in cancer has also been proposed (Lara et al., 2020). Data on the participation of the P2X7R in pathophysiological responses outside the immune system are fewer and less conclusive. We recently described the involvement of the P2X7R in the inflammatory response in intervertebral discs, thus highlighting the need of a more thorough investigation of P2X7R localization and function in cells originating from bone and cartilage (Penolazzi et al., 2022). During this investigation, we realized that in this tissue the P2X7R might be present in non-canonical subcellular localizations.

To date, a rigorous investigation of P2X7R subcellular distribution is still hampered by the lack of highly specific anti-P2X7R antibodies and by the need of well controlled protocols to obtain highly purified subcellular fractions.

Nevertheless, a detailed “subcellular anatomy” of the P2X7R is strongly needed, also in view of the many P2X7R-associated responses that may not directly (or entirely) depend on its channel function, such as changes in plasma membrane lipid symmetry, microparticle shedding, activation of lipases, kinases, and transcription factors, as well as cytokine release and apoptosis (Sluyter, 2017). These many functions might also depend on the peculiar intracellular architecture of this receptor characterized by the presence of a globular domain (the “ballast”) (McCarthy et al., 2019) that accounts for 40% of the entire receptor protein and is responsible for many of the responses typically associated to the P2X7R. More than 50 different putatively P2X7R interacting proteins have been identified so far, but this is at present at the best speculative and additional signal transduction systems besides ion fluxes remain to be defined (Costa-Junior et al., 2011; Kopp et al., 2019). This is mainly due to limited data on the organization and structure of the P2X7R C-terminal tail, post-translational modifications, and spatial organization (subcellular compartmentalization, co-localization or sequestration) of P2X7R and its interaction partners. Importantly, once we know what compartment a protein is in, it is easier to narrow down what it might be doing.

Studies that have so far investigated P2X7R subcellular localization are few. To date, there is evidence suggesting the presence of the P2X7R in the endoplasmic reticulum, lysosomes, and phagosomes and in the mitochondria (Gonnord et al., 2009; Qu and Dubyak, 2009; Sarti et al., 2021). Some of this localization is obvious, due to P2X7R assembly, trafficking to the plasma membrane or proteolytic degradation processes (Barth et al., 2007; Robinson and Murrel-Lagnado, 2013). However, P2X7R localization at the phagosome (Gu and Wiley, 2018) or at mitochondria-associated-membranes (MAMs) (Missiroli et al., 2023) might have a wider pathophysiological meaning in the context of inflammation. Only one paper describes the presence of the P2X7R on the nuclear membrane (Atkinson et al., 2002). Most data on the intracellular localization of the P2X7R derive from experiments in various cell lines, animal models, or immune cells, neurons, glia cells and cancer cells. Although P2X7R expression has been demonstrated in bone cells (osteocytes, osteoclasts, and osteoblasts) (Jørgensen et al., 2002; Agrawal and Gartland, 2015; Dong et al., 2020) and chondrocytes (Tanigawa et al., 2018; Li et al., 2021), the subcellular localization of this receptor in these cells has never been thoroughly investigated. We have previously demonstrated that P2X7R is expressed in primary cultures of both human osteoblasts from bone tissue (Bergamin et al., 2021) and chondrocyte-like cells of the intervertebral disc, the major fibrocartilaginous joint between two vertebrae in the spine (Penolazzi et al., 2022). In the present study, we focused on mapping P2X7R subcellular localization in these cells. *Ex vivo* human primary osteoblasts (OBs), chondrocytes (Chs) and intervertebral disc (IVD) cells from surgical biopsies were used as experimental model. Our data show that the human wild-type full length P2X7R (P2X7A) was expressed in different samples of human osteoblasts, chondrocytes and intervertebral disc cells. Through fluorescence microscopy (LM) and immunogold transmission electron microscopy we localized P2X7R not only in the canonical sites (plasma membrane and cytoplasm), but also in the nucleus of all the 3 cell types, in particular IVD cells and OBs. Mitochondrial P2X7R immunoreactivity was predominantly detected in OBs and in IVD cells, but not in Chs.

## Materials and methods

### Cell isolation and cultures

Human samples were collected after written informed consent provided by the participants and approval of the Ethics Committee of the University of Ferrara and S. Anna Hospital (protocol no. 160998). IVD cells were isolated from human lumbar disc tissues of patients undergoing spinal surgery for herniation and subjected to mild digestion (1 mg/mL type IV collagenase, 5 h, 37 °C) as previously described (Penolazzi et al., 2018). Human osteoblasts (hOBs) were obtained from vertebral lamina discarded during spinal surgery: bone chips were minced into smaller pieces, plated in T-25 culture flasks as previously reported (Lambertini et al., 2017). Human chondrocytes (Chs) were isolated from cartilage of nasal septum after septoplasty surgery procedures (Angelozzi et al., 2017): cartilage fragments were minced into small pieces and rapidly incubated with type IV Collagenase, 16 h, 37 °C (Sigma-Aldrich).

Cells (OBs, IVD cells, and Chs) that were released from the dissected tissue were cultured in standard medium (50% DMEM (Dulbecco’s Modified Eagle Medium) high-glucose/50% DMEM F-12/10% fetal calf serum) (cat. ECB7501L, ECB7502L, Euroclone S. p.A. Milan, Italy) supplemented with antibiotics (penicillin 100 mg/mL and streptomycin 10 mg/mL), at 37 °C in a humidified atmosphere of 5% CO_2_. The cells were then expanded until confluent (passage zero, P0), harvested and used for the experiments here described (passage 2 to passage 4). The morphology and phenotypic characterization of the cells used is reported in the Supplementary Figure S1.

P2X7R negative HEK293 (wild type) and HEK293 stably expressing P2X7A (namely, HEK293-P2X7A) were grown in standard culture medium as above reported (Adinolfi et al., 2010).

See Supplementary Material for detailed information on methodologies.

### Western blotting

Total cell lysates were prepared in lysis buffer (50 mM Tris-HCl, pH 7.8, 1% NP-40, 150 mM NaCl, 0.5% SDC (Sodium Deoxycholate), 0.1% SDS (Sodium Dodecyl Sulfate) and 1 mM NaF) supplemented with protease inhibitors (Sigma-Aldrich). Proteins were quantified using the Bradford protein assay (Bio-Rad Laboratories, Inc., CA, United States) (Bradford, 1976). Thirty micrograms of protein were resolved on NuPAGE Bis-Tris Gel 4%–12% gels (cat. NP0326BOX, Life Technologies), transferred to nitrocellulose membranes and incubated over night at 4 °C with the following primary antibodies: anti-P2X7R C-terminal (cat. APR-004, rabbit anti-human, Alomone labs, Jerusalem, Israel; dilution 1:300 in 2.5% NFDM, Non-Fat Dry Milk); anti-P2X7R extracellular loop (cat. P9122, rabbit anti-human, Sigma Aldrich; dilution 1:300 in 2.5% NFDM); anti-actin antibody (cat. A1978, mouse anti-human, Sigma Aldrich, dilution 1:1000 in 5% NFDM). Nitrocellulose membranes were incubated with the corresponding HRP (Horseradish Peroxidase)-conjugated secondary antibodies (1:3000 dilution in 5% NFDM): goat anti-rabbit (cat. A16096, Life Technologies); goat anti-mouse (cat. 62–6520, Life Technologies).

See Supplementary Material for detailed information on methodologies.

### Immunofluorescence and confocal analysis

Cells (2 × 10^4^) were seeded on glass coverslips put into 24 well plates and fixed in 4% paraformaldehyde for 2 min at 37 °C. After washes, the cells were permeabilized using 0.05% Triton X-100 and then blocked with 2% BSA (Bovine Serum Albumin)/0.05% Triton X-100/PBS (Phosphate Buffered Solution). After that, cells were incubated overnight at 4 °C with the primary antibodies: anti-P2X7R (cat. P8232, C-ter 576–595, rabbit anti-human, 1:100 dilution; Sigma Aldrich) and anti-TOM20 (cat. WH0009804M1, mouse anti human 1:100, Sigma Aldrich). P2X7R blocking peptide (cat. AB5246, Merck KGaA, Darmstadt, Germany) was added to the primary antibody at a 1:1 ratio (Supplementary Figure S2). Appropriate isotype-matched AlexaFluor-conjugated secondary antibodies (diluted 1:1000) were then used (cat. A11008, goat anti-rabbit 488, and cat. A-11003, goat anti-mouse 546, Life Technologies, CA, United States). The coverslips were mounted with ProLong Gold Antifade with DAPI (4′,6-diamidino-2-phenylindole) (cat. P36935, Life Technologies), and immunofluorescence analysis was performed with a confocal laser scanner microscope (Olympus FV3000) equipped with a ×63 oil objective. After background correction, the Mander’s and Pearson’s coefficient for colocalization were analyzed using the JACOP plugin of the open-source Fiji software (http://fiji.sc/Fiji).

### Immunogold labeling and electron microscopy

Cells (2 × 10^6^) were harvested by trypsinization. The cell suspension was fixed in 2% paraformaldehyde/PBS for 1 h, permeabilized with 0.1% Triton X-100 and blocked with PBS/2% BSA (Tao-Cheng et al., 2021). Cells were labelled over night with anti-P2X7R (cat. P8232, C-ter 576–595, rabbit anti-human, 1:20 dilution; Sigma Aldrich) or an equivalent amount of rabbit IgG (Cat. 2729, Cell Signaling Technology, MA, United States), reported as negative control (see Supplementary Figure S3); samples were then incubated with Protein A- 20 nm Colloidal Gold Labeled (cat. P6855, Sigma Aldrich). Finally, cells were fixed in glutaraldehyde 2.5% phosphate buffer and osmium tetroxide 2%, dehydrated and araldite embedded (Sigma-Aldrich). The ultra-thin sections of a selected area were contrasted with uranyl acetate lead citrate, and observed with a Zeiss EM910 transmission electron microscope (ZEISS, Jena, Germany). Images were captured using an Olympus Megaview III digital camera (Olympus Co., Tokyo, Japan). For each cell type the mean percentage of gold particles distribution was quantified (n = 50 random areas) from the extracellular membrane, cytoplasm, nucleus, and mitochondria. Gold particles were manually counted using ImageJ software (http://fiji.sc/Fiji).

### Statistical analysis

All graphs displayed were produced with GraphPad software 8.0 (GraphPad Software Inc., San Diego CA, United States). All the results were expressed as means ± SD from triplicate measurements performed in at least 3 independent experiments.

## Results and discussion

**FIGURE 1:**
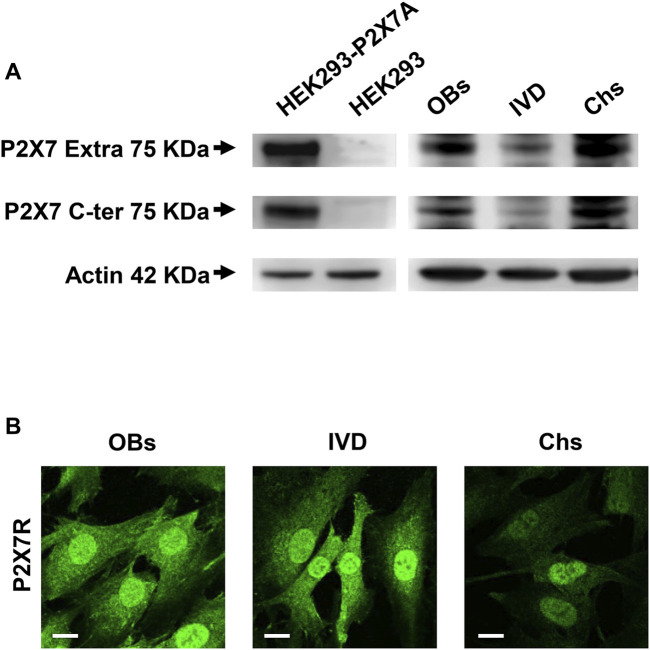
Expression of P2X7R in human osteoblasts (OBs), IVD cells (IVD), and chondrocytes (Chs). **(A)** Representative Western blot analysis with anti-P2X7 extracellular loop and anti-P2X7 C-terminal antibodies; P2X7R stably transfected HEK293 cells (HEK293-P2X7A) and HEK293 wild-type (HEK293) were used as positive and negative control respectively. β-actin was used as loading control. Number of cell samples: OBs = 6, IVD = 4, Chs = 3 **(B)** Representative immunofluorescence and confocal microscopy analysis with anti-P2X7R C-terminal antibody. Scale bars = 10 μm.

The expression of P2X7R was investigated by Western blotting analysis. As shown in Figure 1A, the presence of the human wild-type full length P2X7R (P2X7A) was detected in different samples of human OBs, IVD cells and Chs. P2X7R-transfected HEK293 cells and HEK293 cells (well-known not to express the P2X7R) were used as positive and negative control, respectively. Two different antibodies were used, one raised against the extracellular loop and the other against the intracellular C-terminal domain, as described in the Materials and Methods section. We are well aware that further analyzes will be needed to characterize the presence of possible P2X7R splice variants (e.g., P2X7B) or single nucleotide polymorphisms (SNPs) (Sluyter, 2017) in the different cell populations examined. In any case, the P2X7A protein of the expected size of approximately 75 KDa was identified here by the two antibodies in all the 3 cell types. As reported in Supplementary Figure S4, the same Western blotting analysis highlighted other bands of different molecular weight which, being also present in P2X7R negative HEK293 cells, are considered non-specific.

We then analyzed the 3 cell types by confocal microscopy. All cell types were diffusely stained by the anti-C-terminus antibody, with high intensity in the nucleus. Overall, the signal was higher in OBs and IVD cells than in Chs (Figure 1B).

**FIGURE 2:**
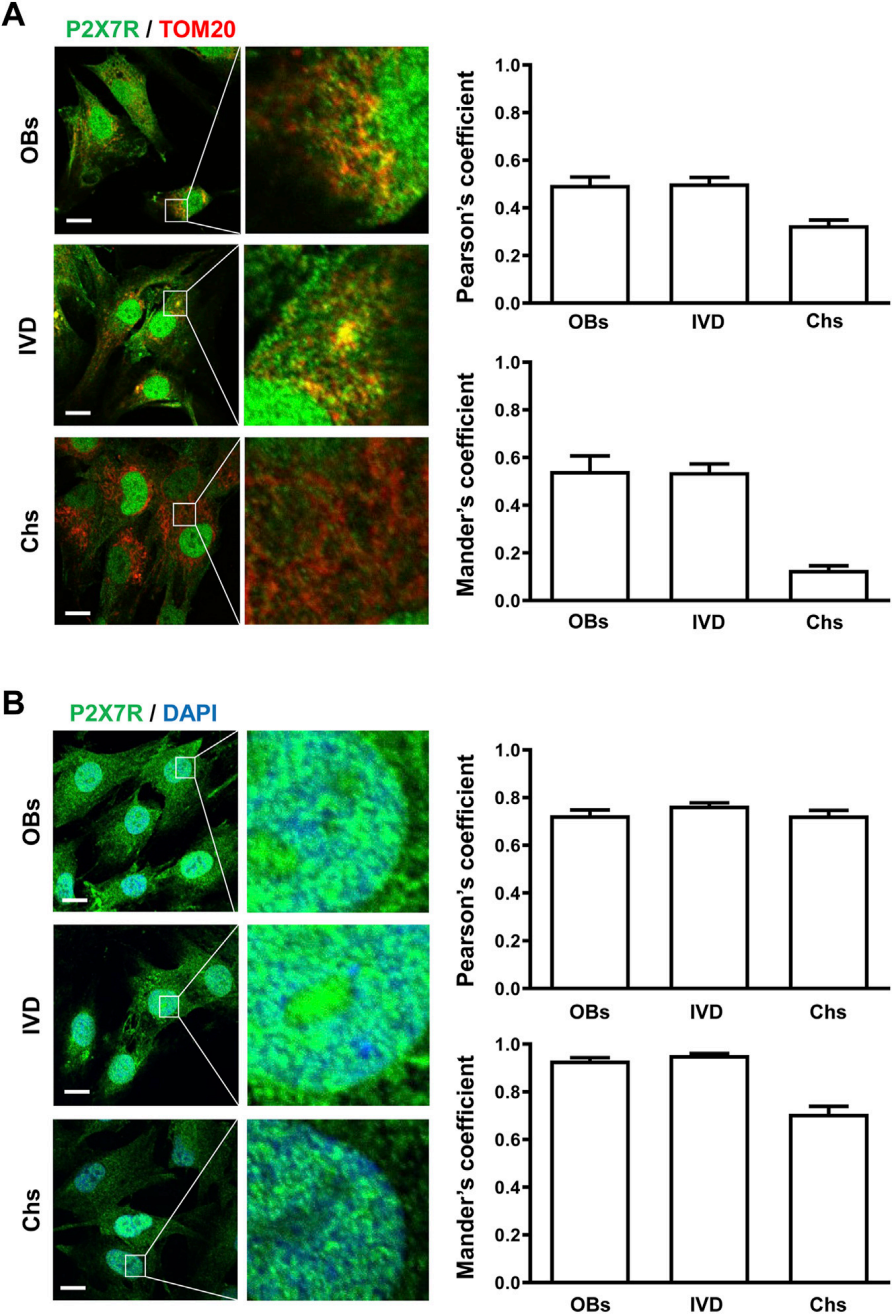
Subcellular localization of P2X7R by immunofluorescence and confocal microscopy in human osteoblasts (OBs), IVD cells (IVD), and chondrocytes (Chs). **(A)** Mitochondrial P2X7R localization analysis. The cells were co-labeled with an anti-P2X7R C-terminal antibody (Alexafluor 488, green) and an anti-TOM20 antibody (Alexafluor 546, red). Merge images represent an overlay of the two channels where co-localization is indicated by a color change (yellow). Average Pearson’s and Mander’s co-localization coefficients (±SEM) were evaluated and reported in the graphs. Scale bars = 10 μm. **(B)** Nuclear P2X7R localization analysis. The cells were co-labeled with an anti-P2X7R C-tail antibody (Alexafluor 488, green) and DAPI (nuclear staining, blue). Co-localization of P2X7R with DAPI was assessed by Pearson’s and Mander’s coefficients (average ± SD). Scale bars = 10 μm.

To analyze the P2X7R subcellular localization we immunolocalized the P2X7R by either fluorescence microscopy (LM) or immunogold transmission electron microscopy (TEM). Given structural and functional changes observed during osteogenic and chondrogenic differentiation in the mitochondria and the nucleus (Li et al., 2017; Goelzer et al., 2021), we focused on these two organelles. Mitochondrial P2X7R localization was evaluated by co-labeling with the anti-P2X7R C-tail antibody and an anti-TOM20 antibody (Translocase of the Outer Membrane, a general import receptor that recognizes mitochondrial targeting signals) (Yano et al., 2004). As reported in Figure 2 A, immunofluorescence showed that P2X7R co-localized with TOM20 both in OBs and IVD cells, as revealed by Pearson’s and Manders’ overlap coefficient. On the contrary, Chs showed a low co-localization index (PC < 0.3, MC < 0.2), suggesting the absence of P2X7R in the mitochondria.

Co-labeling of cells with anti-P2X7R C-tail antibody and DAPI for nuclear staining (Figure 2B) confirmed the presence of P2X7R in the nucleus of all the cells examined.

**FIGURE 3:**
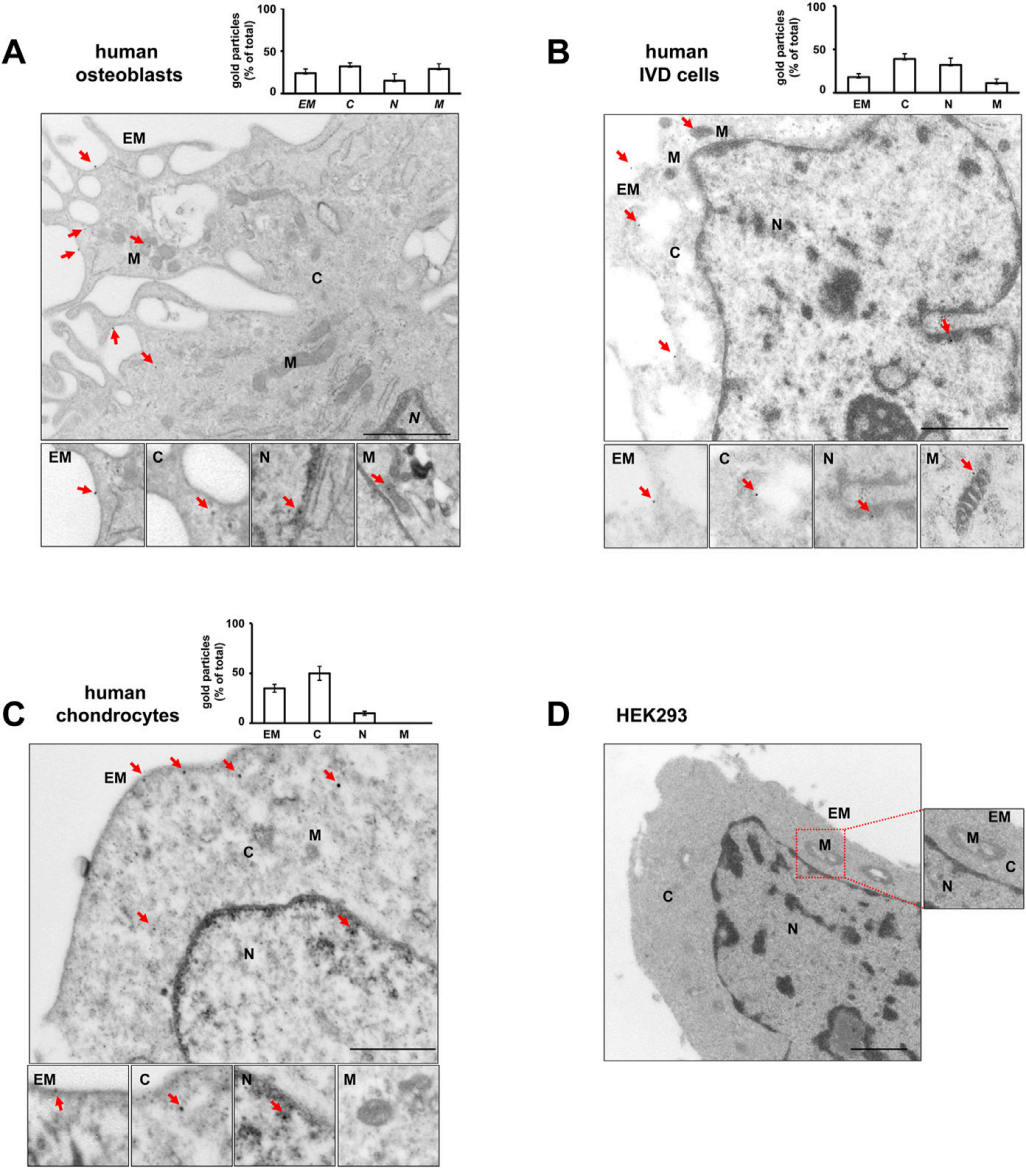
Subcellular localization of P2X7R by immunogold labeling. Human osteoblasts **(A)**, IVD cells **(B)**, chondrocytes **(C)** and HEK293 wild-type cells **(D)** were subjected to pre-embedding immunogold staining. For each cell type the mean percentage of gold particles distribution was reported. Fifty areas from the extracellular membrane (*EM*), cytoplasm (*C*), nucleus (*N*) and mitochondria (*M*) were randomly chosen, and gold particles were manually counted using ImageJ software, and expressed as percent of total (±SD). High magnification boxes for each subcellular compartment were reported below. Arrowheads = gold particles. Scale bars = 1 µm.

High-resolution visualization of P2X7R distribution by TEM revealed P2X7R immunoreactivity (red arrows in Figure 3) not only in the canonical sites, i.e., plasma membrane and cytoplasm, but also in the nucleus of all the 3 cell types, OBs, IVD cells, and Chs (Figures 3A,B,C). Nuclear P2X7R immunoreactivity appeared to be prevalent in IVD cells, whereas mitochondrial P2X7R immunoreactivity appears to be predominant in OBs. In the Chs P2X7R was mainly localized in the plasma membrane and cytoplasm, very little in the nucleus, and none in the mitochondria, in agreement with data from immunofluorescence confocal microscopy.

In control experiments, no gold labelling was seen in P2X7R negative HEK293 cells (Figure 3D) nor in cells labelled with rabbit IgG (Supplementary Figure S3). P2X7R immunoreactivity was predominantly detected in the plasma membrane of P2X7R-transfected HEK293 cells (Supplementary Figure S5).

These data suggest that P2X7R may be considered a protein with multiple localizations. This is not so surprising considering that about a half of all proteins are localized at multiple compartments, and that there is a shared pool of proteins even among functionally unrelated organelles as demonstrated by recent spatial proteomics approaches (Thul et al., 2017). From the data present in the literature to date, it does not appear that P2X7R is equipped with the typical sorting signals of subcellular localizations (Kopp et al., 2019; Sarti et al., 2021). We subjected the P2X7R sequence to computational analysis (Imai and Nakai, 2020) to identify putative mitochondrial and nuclear localization signals, but we found no canonical mitochondrial localization signals. Most likely P2X7R is part of the 40% of mitochondrial proteins lacking the sequence for subcellular localization (Bauer et al., 2015). It has been in fact reported that mitochondrial proteins that are synthesized without a cleavable presequence typically recognized by the translocase of the outer membrane (TOM) receptors, may also enter mitochondria via unusual pathways (Chacinska et al., 2009; Jackson et al., 2021). On the contrary, and very interestingly, NLStradamus tool (Nguyen Ba et al., 2009) revealed the presence of a nuclear localization signal at Cter of P2X7R (578 - RKEFPK - 583).

It is conceivable that P2X7R uses some alternative pathways based on molecular adaptors or protein scaffolds capable of modulating the localization of the receptor and consequently its function, all aspects that certainly deserve to be investigated in detail. It is important to highlight these issues because the design of novel P2X7R-targeted therapies will have to take them into account (Burnstock, 2006). The unorthodox compartmentalization of P2X7R in cells of the skeletal system might be functional to:

- A) modulate its activation state and stability
- B) promote its degradation, turnover or recycling via different pathways
- C) integrate the P2X7R in different intracellular circuitries, intracellular organelle exchange, or specific metabolic pathways or intracellular second messenger systems (e.g., Ca^2+^ signals) (Di Virgilio et al., 2022)
- D) support non-canonical nuclear ATP-generating systems (Wright et al., 2016)
- E) support nuclear mechano-transduction (Kong et al., 2021).

These tips might help to resolve many controversial aspects regarding the role of P2X7R in the cartilage and bone tissues. A large scientific literature has demonstrated that the protection of articular cartilage and the maintenance of joint extracellular matrix homeostasis, as well as the fine-tuning, initiation and termination of balanced bone remodeling are strongly dependent on the purinergic signaling, but many aspects remain to be understood (Corciulo and Cronstein, 2020; Bolamperti et al., 2022). Importantly, the more we know about how mechanical signals are transduced and regulate cell functioning, the better we can understand the different behaviors of the cells present in the joint and bone microenvironment. This knowledge might be of great help for the design of novel therapies for osteoarticular diseases.

## Data Availability

The raw data supporting the conclusion of this article will be made available by the authors, without undue reservation.
