# Supplementary figures and images for: Unorthodox localization of P2X7 receptor in subcellular compartments of skeletal system cells
Source: Front Cell Dev Biol. 2023 May 4;11:1180774. doi: 10.3389/fcell.2023.1180774 (PMC10192554; doi:10.3389/fcell.2023.1180774)

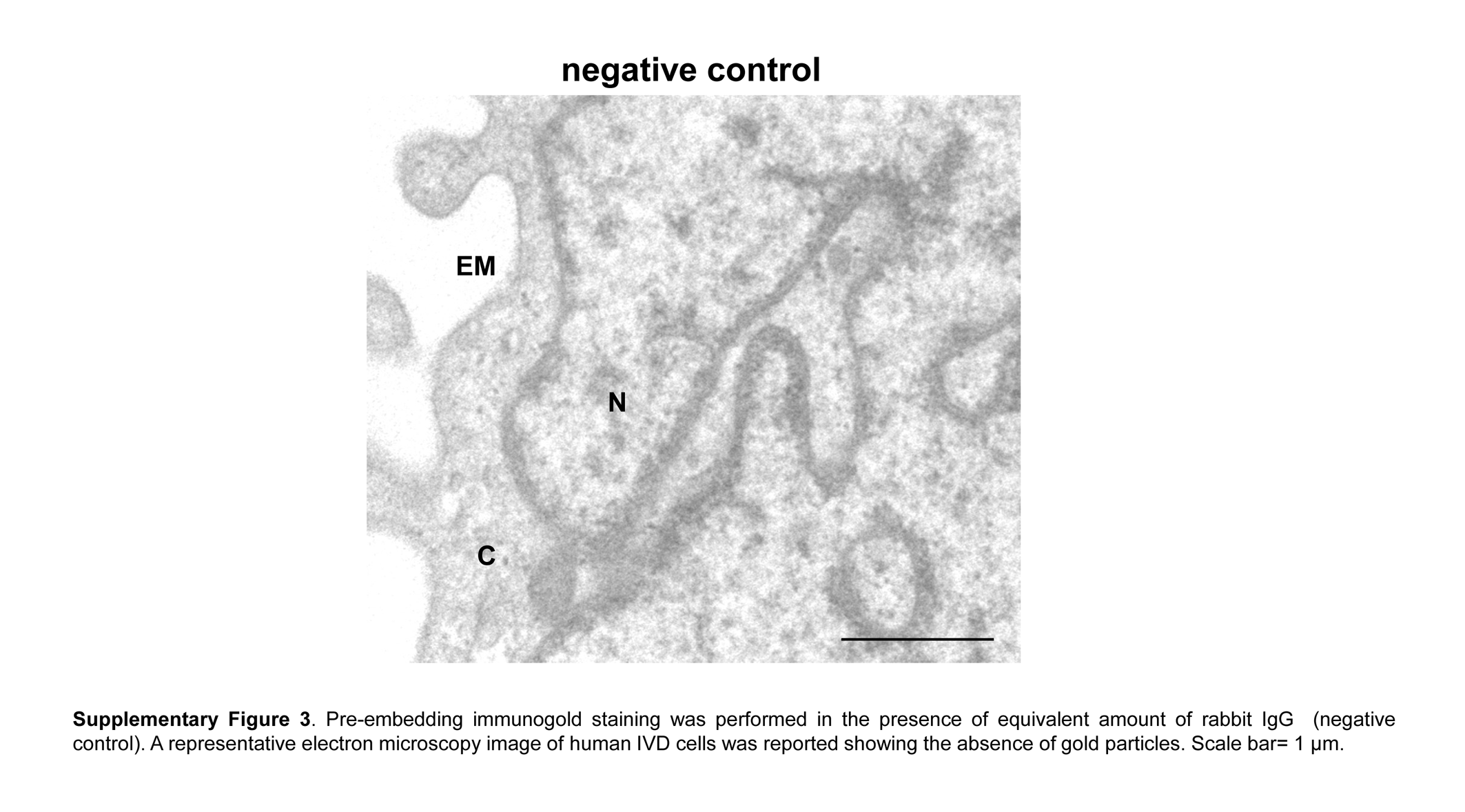

Supplement: Supplementary file 1 [file Image3.TIF]

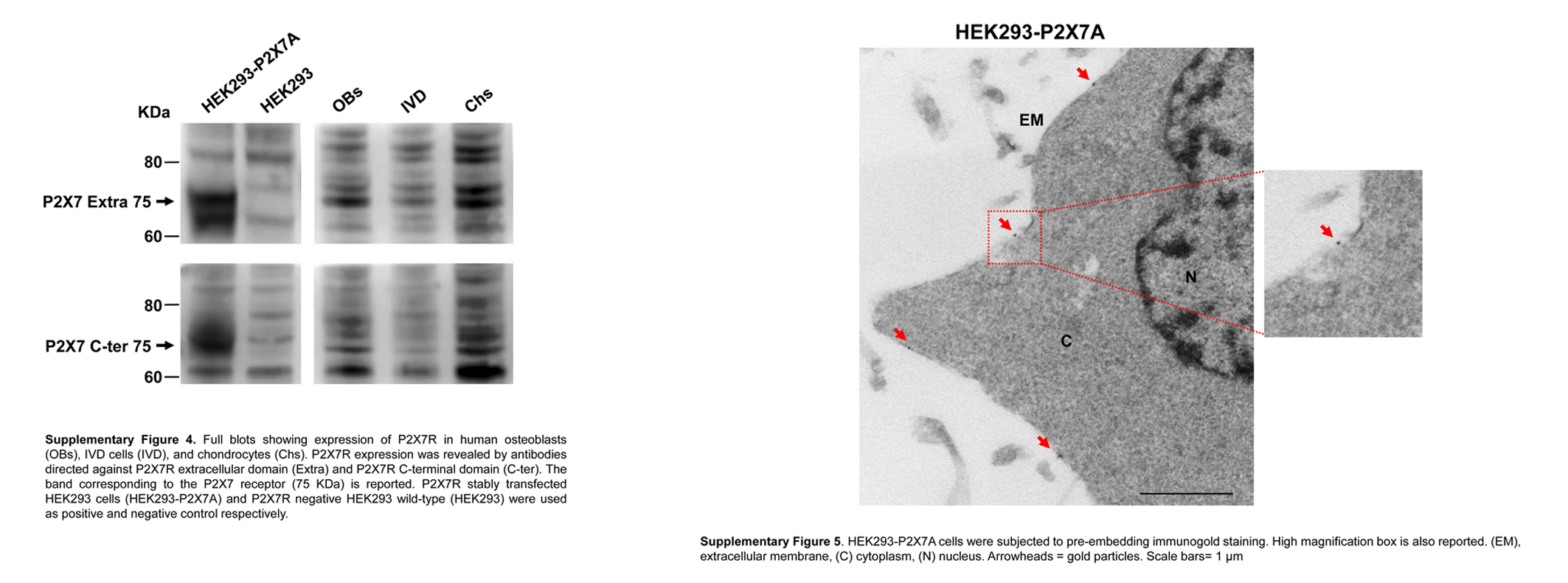

Supplement: Supplementary file 2 [file Image4.TIF]

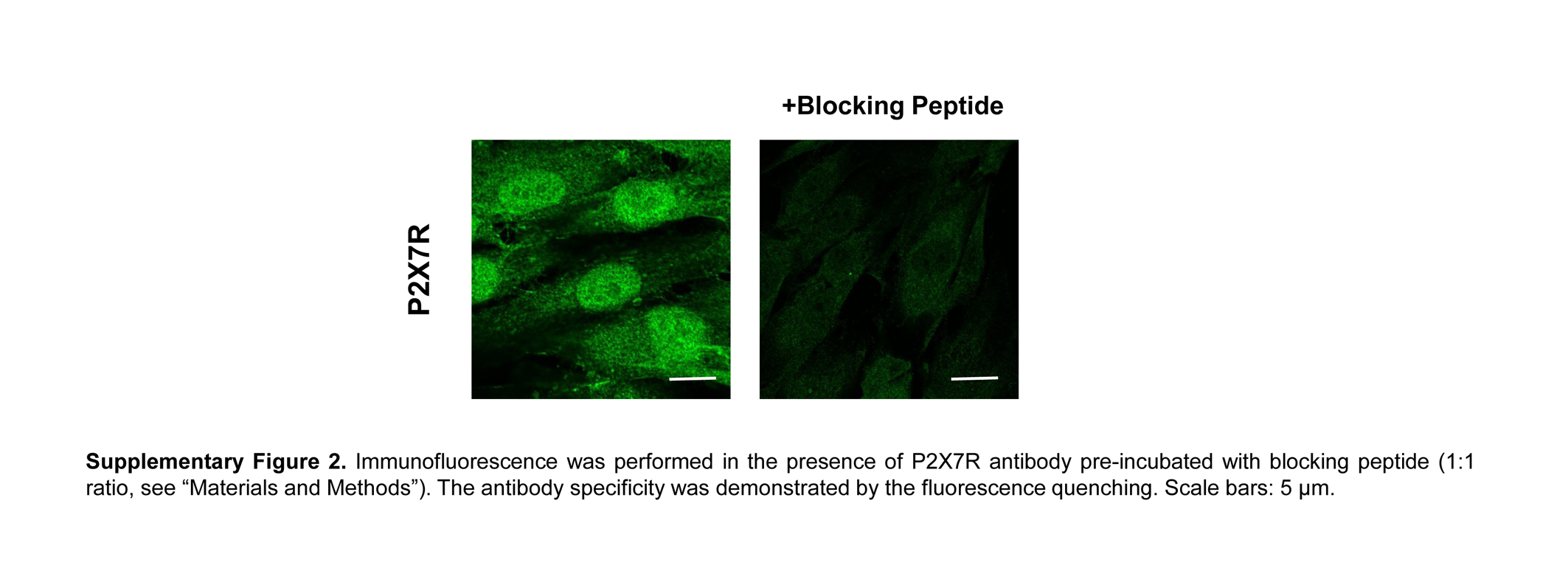

Supplement: Supplementary file 3 [file Image2.TIF]

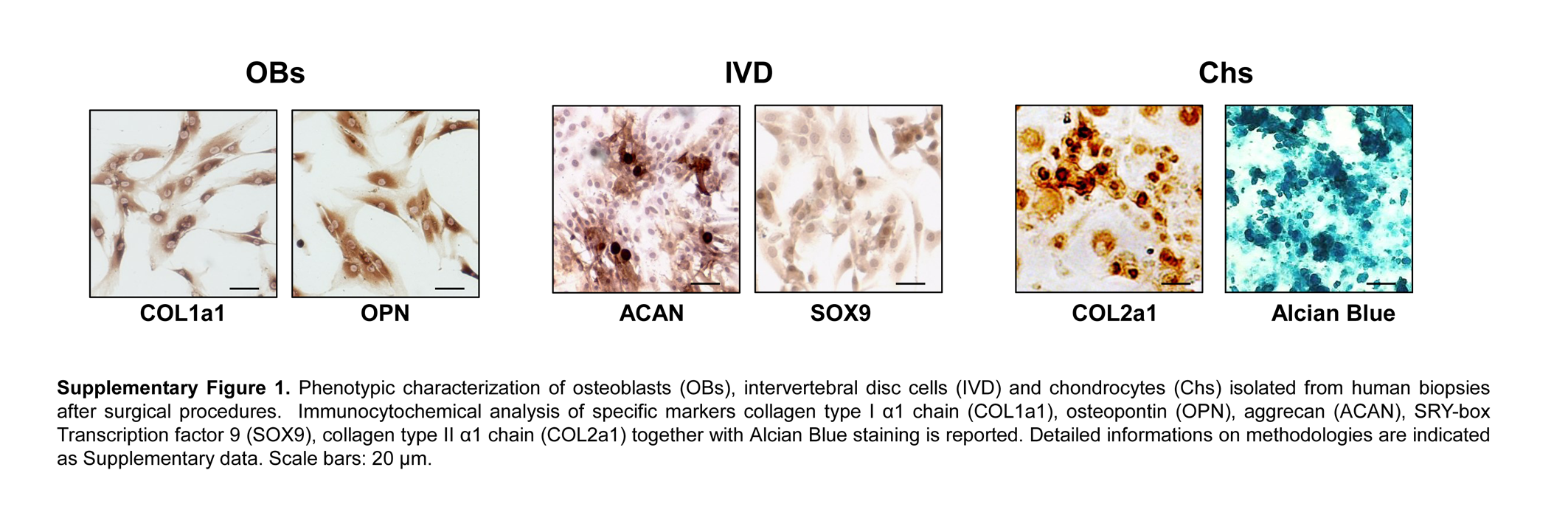

Supplement: Supplementary file 4 [file Image1.TIF]
