# Supplementary material for: Unorthodox localization of P2X7 receptor in subcellular compartments of skeletal system cells
Source: Front Cell Dev Biol. 2023 May 4;11:1180774. doi: 10.3389/fcell.2023.1180774 (PMC10192554; doi:10.3389/fcell.2023.1180774)
Supplement: Supplementary file 5 [file DataSheet1.PDF]

Materials and methods – detailed information

Cell isolation and cultures

Human samples were collected after written informed consent provided by the participants and approval of the Ethics Committee of the University of Ferrara and S. Anna Hospital (protocol no. 160998).
IVD cells were isolated from human lumbar disc tissues of patients undergoing surgical discectomy. The mean age of the donors was 49 years (range, 40-63 years, 2 males and 2 females, Pfirrmann grade 3-5). Nucleus pulposus tissue from each sample was macroscopically dissected from the annulus fibrosus and was subjected to enzymatic digestion using 1 mg/ml type IV collagenase (Sigma-Aldrich; Merck KGaA) for 5 h at 37˚C in DMEM (Dulbecco's Modified Eagle Medium)/Ham's F12. Once the digestion was terminated, the cell suspension was filtered through a Falcon™ 70 µm Nylon Cell strainer (BD Biosciences). Subsequently, the cells were centrifuged at 300 x g for 10 min at room temperature, the supernatant was discarded, the cells were resuspended in basal medium [DMEM/F12 containing 10 % fetal calf serum (FCS; Euroclone S.p.A), 100 mg/ml streptomycin, 100 U/ml penicillin and 1% Glutamine] and seeded in polystyrene culture plates (Sarstedt) at a density of 10000 cells/cm^2 and subcultured up to passage 3.
Human osteoblasts (hOBs) were obtained from vertebral lamina discarded during spinal surgery (n=6, mean age 51, range 40-65 years, 3 females and 3 males). The bone specimens were placed in sterile phosphate buffered solution (PBS) at 4°C and were dissected within 16 h after removal. Bone chips were minced in small fragments (2 mm^3) as previously reported (Lambertini et al., 2017), washed twice with PBS 1X, placed in T25 culture flasks (Sarstedt) and cultured in 50% DMEM high glucose/50% DMEM F12 supplemented with 20% fetal calf serum (Euroclone) and 100 µg/ml streptomycin/100 U/ml penicillin (Sigma Aldrich, St. Louis) at 37°C, in a 5% CO_2 humidified atmosphere. Upon detection of a cell colony from the bone fragments (after 5–8 days) the cells were expanded until confluent (passage zero). Then the cells were detached by trypsinization (0.05% Trypsin–EDTA solution, Sigma Aldrich) harvested, washed, counted with a hemocytometer, and plated at the confluence of 10000 cells/cm^2 for further in vitro experiments (passage 1 to passage 3).
Cartilage fragments from nasal septum were obtained from 3 donors between 30 and 60 years (mean age 45 years), which underwent septoplasty surgery procedures. Briefly, cartilage fragments were minced into small pieces and rapidly 1 mg/ml type IV collagenase (Sigma-Aldrich) for 16 h at 37˚C in DMEM/Ham's F12. Cells were harvested by centrifugation and plated (p0) at a density of 20000 cells/cm^2 in tissue culture flasks (25 cm^2) in standard medium (50% DMEM high-glucose/50% DMEM Ham's F-12/10% fetal calf serum) (Euroclone) supplemented with antibiotics (penicillin 100 mg/mL and streptomycin 10 mg/mL), at 37°C in a humidified atmosphere of 5% CO_2. After 7 days, the culture medium was removed and then changed twice a week. At 70–80% confluence, were detached by trypsinization (0.05% Trypsin–EDTA solution, Sigma Aldrich) harvested, washed, counted with a hemocytometer, and plated at the confluence of 10000 cells/cm^2 for further in vitro experiments (passage 1 to passage 3).
P2X7R negative HEK293 (wild type) and HEK293 stably expressing P2X7A (namely HEK293-P2X7A) were grown in standard culture medium as above reported (Adinolfi et al., 2010).

Immunocytochemical analysis and Alcian Blue staining

Immunocytochemical analysis was performed employing the ImmPRESS kit (cat. MP-7500; Vectorlabs, Burlingame, CA). 2x10^4 cells (hOBs, IVD and Chs) were grown in culture plates, fixed in cold 100% methanol and permeabilized with 0.2% (v/v) Triton X-100 (Sigma-Aldrich) in TBS 1X (Tris-buffered saline). Cells were treated with 3% H_2O_2 in TBS 1X, and incubated in 2% normal horse serum (Vectorlabs) for 15 min at room temperature. After the incubation in blocking serum, primary antibodies (1:200 dilution) were added and incubated overnight (4°C): Collagen type I α1 chain (COL1a1, cat. sc-28657), aggrecan (ACAN, cat. sc-33695), SRY-box Transcription factor 9 (SOX9, cat. sc-20095), from Santa Cruz Biotechnology, Inc., TX, USA; Collagen type II α1 chain (COL2a1, cat. ab3092), from Abcam, Cambridge, UK; osteopontin (OPN, LF-123) is a generous gift from dr L. Fisher (NIH, Bethesda, USA). After rinsing in TBS 1X, the cells were incubated for 30 min at room temperature with ImmPRESS reagent and then stained with substrate/chromogen mix (ImmPACT™ DAB). After washing the cells were mounted in glycerol/PBS (9:1) and observed with a Nikon Eclipse 50i optical microscope (Nikon Corporation, Tokyo).
Human chondrocytes (Chs) were subjected to Alcian Blue staining. Briefly cells were rinsed with PBS 1X, fixed in 4% paraformaldehyde for 10 min and then stained with Alcian Blue pH 2 (1% in 3% acetic acid) (Sigma-Aldrich) overnight at 37°C. The day after, cells were rinsed with PBS 1X and observed with a Nikon Eclipse 50i optical microscope (Nikon Corporation). The presence of GAG deposits appeared as blue staining areas.

Western blotting

For western blotting analysis, cells were washed with ice-cold PBS 1X and lysed with ice-cold RIPA buffer (50 mM Tris-HCl, pH 7.8, 1% NP-40, 150 mM NaCl, 0.5% SDC (Sodium Deoxycholate), 0.1% SDS (Sodium Dodecyl Sulfate) and 1 mM NaF) supplemented with protease inhibitors (Sigma-Aldrich). Lysates were kept on ice for 30 min and centrifuged at 12000 x g for 10 min at 4˚C. Protein concentration was quantified using the Bradford protein assay (Bio-Rad Laboratories, Inc., CA, USA) with BSA (Bovine Serum Albumin, Sigma-Aldrich) as the standard. Thirty micrograms of protein were resolved on NuPAGE Bis-Tris Gel 4–12% gels (cat. NP0326BOX, Life Technologies), transferred to nitrocellulose membranes. Nonspecific binding was blocked with 5% (w/v) NFDM (Non-Fat Dry Milk) in TBS-0.1% Tween-20 (TBST) for 1 h at room temperature, followed by incubation with primary anti-P2X7R C-terminal antibody (cat. APR-004, rabbit anti-human, Alomone labs, Jerusalem, Israel; dilution 1:300 in 2.5% NFDM) overnight at 4°C. Membranes were washed with TBST and incubated with Horseradish Peroxidase -conjugated goat anti-rabbit IgG antibody (cat. A16096, Life Technologies; dilution 1:3000 in 5% NFDM) for 1 h at room temperature. Membranes were stripped using RESTORE Western blot stripping buffer (cat. 21059, ThermoFisher Scientific) for 15 min at 37°C, washed several times, and blocked for 1 h. Then membranes were incubated with primary anti-P2X7R extracellular loop antibody (cat. P9122, rabbit anti-human, Sigma Aldrich; dilution 1:300 in 2.5% NFDM), followed by 1:3000 dilution of HRP-conjugated goat anti-rabbit IgG antibody (1 h at room temperature). A mouse anti-actin antibody (cat. A1978, Sigma Aldrich, dilution 1:1000 in 5% NFDM) was used as loading control and the membranes were incubated with HRP-conjugated goat anti-mouse IgG antibody (cat. 62-6520, Life Technologies;

dilution 1:3000 in 5% NFDM) for 1 h at room temperature. Protein expression was detected using Immobilon Western Chemiluminescent HRP Substrate (cat. WBKLS0500; Millipore Sigma).

Immunofluorescence and confocal analysis

Cells ($2x10^4$) were seeded on glass coverslips put into 24 well plates and fixed in 4% paraformaldehyde for 2 min at 37°C. After three washes with PBS 1X, the cells were permeabilized using 0.05% (vol/vol) Triton X-100 in PBS 1X for 10 min; then cells were incubated in the blocking solution containing 2% BSA/0.05% Triton X-100 in PBS 1X for 40 min. After that, cells were incubated overnight at 4°C with the primary antibodies: anti-P2X7R (cat. P8232, C-ter 576-595, rabbit anti-human, 1:100 dilution; Sigma Aldrich) and anti-TOM20 (cat. WH0009804M1, mouse anti human 1:100, Sigma Aldrich). Where required P2X7R blocking peptide (cat. AB5246, Merck KGaA, Darmstadt, Germany) was added to the primary antibody at a 1:1 ratio (Supplementary Figure 2). Then, cells were incubated with appropriate isotype-matched AlexaFluor-conjugated secondary antibodies (diluted 1:1000) (cat. A11008, goat anti-rabbit 488, and cat. A-11003, goat anti-mouse 546, Life Technologies, CA, USA) in 2% BSA/0.05% Triton X-100 in PBS 1X for 1 h at RT. Cells were then washed three times with 0.1% Triton X-100 in PBS. The coverslips were mounted with ProLong Gold Antifade with DAPI (4',6-diamidino-2-phenylindole) (cat. P36935, Life Technologies), and immunofluorescence analysis was performed with a confocal laser scanner microscope (Olympus FV3000) equipped with a 63X oil objective. After background correction, the Mander's and Pearson's coefficient for colocalization were analyzed using the JACOP plugin of the open-source Fiji software (http://fiji.sc/Fiji).

Immunogold labeling and electron microscopy

Cells ($2x10^6$) were detached by trypsinization, harvested by centrifugation and fixed in 2% paraformaldehyde/PBS 1X for 1 h at room temperature. The cell suspension was then permeabilized with 0.1% Triton X-100 and blocked with 2% BSA in PBS 1X (Tao-Cheng et al., 2021). Cells were labelled over night (4°C) with anti-P2X7R (cat. P8232, C-ter 576-595, rabbit anti-human, 1:20 dilution; Sigma Aldrich) diluted in 0.2% BSA in PBS 1X or an equivalent amount of rabbit IgG (cat. 2729, Cell Signaling Technology, MA, USA), reported as negative control (see Supplementary Figure 3). After washes in PBS 1X, cells were incubated with Protein A - 20 nm Colloidal Gold Labeled diluted 1:20 in 0.2% BSA in PBS 1X (cat. P6855, Sigma Aldrich). Finally, cells were fixed in glutaraldehyde 2.5% phosphate buffer and osmium tetroxide 2%, dehydrated and araldite embedded (Sigma-Aldrich). The ultra-thin sections of a selected area were contrasted with uranyl acetate lead citrate, and observed with a Zeiss EM910 transmission electron microscope (ZEISS, Jena, Germany). Images were captured using an Olympus Megaview III digital camera (Olympus Co., Tokyo, Japan). For each cell type the mean percentage of gold particles distribution was quantified (n=50 random areas) from the extracellular membrane, cytoplasm, nucleus, and mitochondria. Gold particles were manually counted using ImageJ software (http://fiji.sc/Fiji).

Statistical analysis

All graphs displayed were produced with GraphPad software 8.0 (GraphPad Software Inc., San Diego CA, USA). All the results were expressed as means ± SD from triplicate measurements performed in at least 3 independent experiments.
